# A Multiomics Profiling Based on Online Database Revealed Prognostic Biomarkers of BLCA

**DOI:** 10.1155/2022/2449449

**Published:** 2022-05-25

**Authors:** Hanwen Li, Shaohua Chen, Hua Mi

**Affiliations:** ^1^Department of Urology, The First Affiliated Hospital of Guangxi Medical University, Nanning, China; ^2^Institute of Urology and Nephrology, The First Affiliated Hospital of Guangxi Medical University, Nanning, China; ^3^Guangxi Collaborative Innovation Center for Genomic and Personalized Medicine, Nanning, China; ^4^Guangxi Key Laboratory for Genomic and Personalized Medicine, Nanning, China; ^5^Guangxi Key Laboratory of Colleges and Universities, Nanning, China

## Abstract

**Background:**

Bladder cancer (BLCA) is one of the most common urological malignancies globally, posing a severe threat to public health. In combination with protein-protein interaction (PPI) network analysis of proteomics, Gene Set Variation Analysis (GSVA) and “CancerSubtypes” package of *R* software for transcriptomics can help identify biomarkers related to BLCA prognosis. This will have significant implications for prevention and treatment.

**Method:**

BLCA data were downloaded from The Cancer Genome Atlas (TCGA) database and GEO database (GSE13507). GSVA analysis converted the gene expression matrix to the gene set expression matrix. “CancerSubtypes” classified patients into three subtypes and established a prognostic model based on differentially expressed gene sets (DEGSs) among the three subtypes. For genes from prognosis-related DEGSs, functional and pathway enrichment analyses and PPI network analysis were carried out. The Human Protein Atlas (HPA) database was used for validation. Finally, the proportion of tumor-infiltrating immune cells (TIICs) was determined using the CIBERSORT algorithm.

**Results:**

In total, 414 tumor samples and 19 adjacent-tumor samples were obtained from TCGA, with 145 samples belonging to subtype A, 126 samples belonging to subtype B, and 136 samples belonging to subtype C. Then, we identified 83 DEGSs and constituted a prognostic signature with two of them: “GSE1460_CD4_THYMOCYTE_VS_THYMIC_STROMAL_CELL_DN” and “MODULE_253.” Finally, five subnets of two PPI networks were established, and nine core proteins were obtained: CDH2, COL1A1, EIF2S2, PSMA3, NAA10, DNM1L, TUBA4A, KIF11, and KIF23. The HPA database confirmed the expression of the nine core proteins in BLCA tissues. Furthermore, EIF2S2, PSMA3, DNM1L, and TUBA4A could be novel BLCA prognostic biomarkers.

**Conclusions:**

In this study, we discovered two gene sets linked to BLCA prognosis. PPI analysis confirmed the network's core proteins, and several newly discovered biomarkers of BLCA prognosis were identified.

## 1. Introduction

Bladder cancer (BLCA) is one of the most common urological malignancies globally and a leading cause of cancer deaths [1]. According to studies, the incidence of bladder cancer has been increasing in recent years [2]. This implies that BLCA poses a serious threat to public health. According to epidemiological studies, cigarette smoking is the leading risk factor for BLCA; however, tobacco cessation interventions do not appear clinically effective for mortality [3]. The mechanisms underlying the phenomena must be understood. As a heterogeneous tumor, BLCA has variable prognosis influenced by different subtypes. BLCA is composed of two major subtypes: nonmuscle-invasive bladder cancer (NMIBC) and muscle-invasive bladder cancer (MIBC) [4]. Most patients are confirmed with NMIBC confined to the mucosa or lamina propria, which is associated with a better prognosis.

Meanwhile, 25% of patients have MIBC that invades the detrusor muscle and also has the potential to metastasize to lymph nodes or distant organs [5, 6]. These differences could account for the large prognostic gap between NMIBC and MIBC. BLCA can also be classified into several subtypes based on tissue morphology phenotypes, including urothelial carcinoma, squamous cell carcinoma, and adenocarcinoma [7], but the effect of tissue morphology phenotypes on prognosis remains debated [8].

Based on transcriptomic data from The Cancer Genome Atlas (TCGA) database, bioinformatics analysis can reclassify BLCA more reasonably and reveal the mechanism different underlying prognosis. To analyze TCGA RNA-seq data, we used gene set variation analysis (GSVA), a nonparametric unsupervised method. GSVA could be used to assess the gene expression at the pathway level rather than at the gene level. This method outperforms single-gene analysis in terms of feature dimension and noise interference, as well as biological interpretability [9]. Furthermore, the “Cancersubtypes” *R* software package has been developed to reveal molecular subtypes of cancer patients from public databases using multiomics data: gene expression, DNA methylation, and miRNA expression [10]. This *R* package has recently been used in several human cancer studies [11–13], demonstrating the feasibility of this new method. However, no studies have been conducted to classify BLCA using the “Cancersubtypes” package. Protein-protein interaction (PPI) network analysis could build a protein network and then analyze their interactions [14]. In this study, RNA-seq data from the TCGA-BLCA cohort was analyzed by bioinformatics using GSVA and “Cancersubtypes.” The results were combined with proteomic analysis to reach a more convincing conclusion. Exploring the potential mechanisms influencing prognosis among different BLCA subtypes and the prognostic marker is critical for improving BLCA patient survival outcomes.

## 2. Materials and Methods

### 2.1. Data Collection and Processing

Transcriptome data and clinical characteristics from BLCA patients were obtained from the TCGA database (https://portal.gdc.cancer.gov/) and used as a training set. The testing set was composed of GSE13507 datasets obtained from the GEO database (https://www.ncbi.nlm.nih.gov/geo/). For further analysis, only samples with complete prognostic data were extracted.

### 2.2. Gene Set Variation Analysis

The GSVA algorithm was used with *R* language's “GSVA” package to reveal biological correlations between training and testing set genes. GSVA is a nonparametric, unsupervised method for identifying closely related pathways to essential genes [9]. By inputting gene expression matrix of training and testing sets, and a collection of gene sets, including “hallmark gene sets,” “KEGG subset of Canonical pathways,” and “immunologic signature gene sets” downloaded from the GSEA database (http://www.gsea-msigdb.org/gsea/index.jsp) for GSVA analysis, the gene expression matrix could be transformed to the matrix of gene set expression to explain the corresponding biological meaning.

### 2.3. Identification of BLCA Subtypes

The *R* package “CancerSubtypes” helps infer cancer subtypes from input gene sets, using a consensus clustering algorithm to determine the number of subtypes, and uncover potential differences among varying subtypes [10]. The relationship between clinical characteristics (age, gender, grade, stage, TNM stage) and classification was investigated. The *R* software's “limma” package was used to identify DEGSs within each subtype. DEGSs with a |log2 fold change| of 0.1 and an adjusted *P* < 0.05 were excluded from further analysis.

### 2.4. Construction of BLCA Prognostic Signature

Univariate Cox analysis and Kaplan–Meier survival analysis were performed in *R* software using the “survival” and “survminer” packages to obtain prognostic DEGSs. Then, using the “glmnet” *R* package, LASSO Cox regression analysis was used to establish the BLCA prognostic signature. The associated expression of these prognostic DEGSs and their correlated coefficient were used to calculate the risk score (RS) of the prognostic DEGSs for each sample. The median value of RS was used to group BLCA samples.

### 2.5. Functional and Pathway Enrichment Analysis

Pathways with gene symbols were downloaded from KEGG (https://www.kegg.jp/kegg/) and GO (http://geneontology.org/). Functional and pathway enrichment analysis on these prognosis-related genes was performed using the *R* packages “Cluserprofiler.”

### 2.6. Establishment of Protein-Protein Interaction Network

The PPI network was created using the Search Tool for the Retrieval of Interacting Genes (STRING) database (https://string-db.org/). Furthermore, the Cytoscape software was used to search for meaningful modules in the PPI network (degree cutoff = 2, maximum depth = 100, *k* − core = 2, and node score cutoff = 0.2).

### 2.7. Validation of the Core Protein Expression

Several core proteins were chosen from the PPI network. We used the HPA database (http://www.proteinatlas.org/) to obtain the expression level of selected core proteins for further validation.

### 2.8. Tumor-Infiltrating Immune Cell (TIICs) Analysis

The CIBERSORT algorithm was used to estimate the composition of 22 immune cells in the BLCA prognostic signature between high- and low-RS groups. The relative abundance of each immune cell was calculated based on the gene expression.

### 2.9. Statistical Analysis

All statistical analyses were conducted using *R* v4.0.3 (https://www.r-project.org/) and Perl v5.32.1.1 (https://strawberryperl.com/). OS was defined as the time between intervals from the date of diagnosis and the date of death by any cause. The prognostic value of gene sets was assessed by Cox regression analysis. *P* < 0.05 was considered statistically significant.

## 3. Results

### 3.1. The GSVA Analysis for BLCA

**Figure 1:**
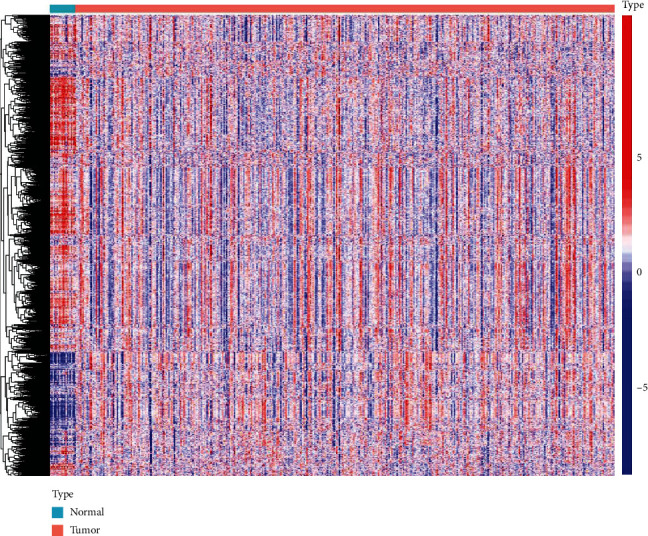
Heat map of the BLCA gene set expression in the TCGA database. The color blue to red represents the expression level from low to high.

Four hundred thirty-three samples were obtained from the TCGA, including 414 tumor samples and 19 adjacent-tumor samples. Using the *R* package “GSVA,” these sample gene expression matrices were subjected to the GSVA algorithm of hallmark gene sets, KEGG subsets of canonical pathways, and immunologic signature gene sets. We also conducted the GSVA algorithm in GEO datasets GSE13507 for subsequent validation. This step condenses gene-level RNA-seq expression profiles into gene sets used in subsequent analyses. The expression of gene sets in TCGA samples is depicted as a heat map (Figure 1).

### 3.2. Identification of BLCA Subtypes

**Figure 2:**
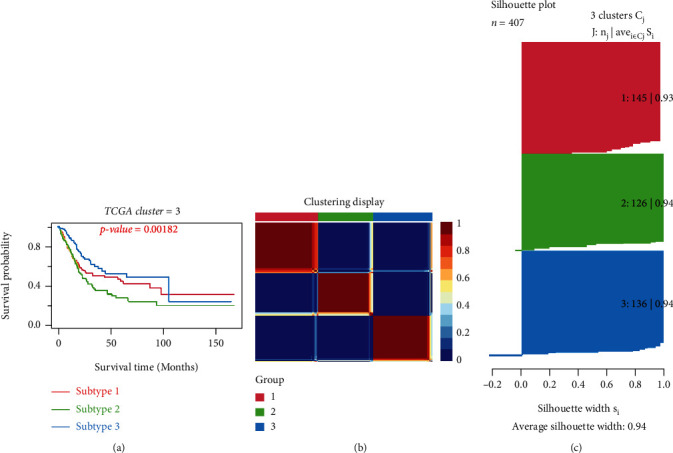
Classification of BLCA subtypes using “CancerSubtypes” in the TCGA. (a) Kaplan–Meier survival analysis of three subtypes. (b) Clustering heat map. (c) Average silhouette width represents the coherence of clusters.

**Figure 3:**
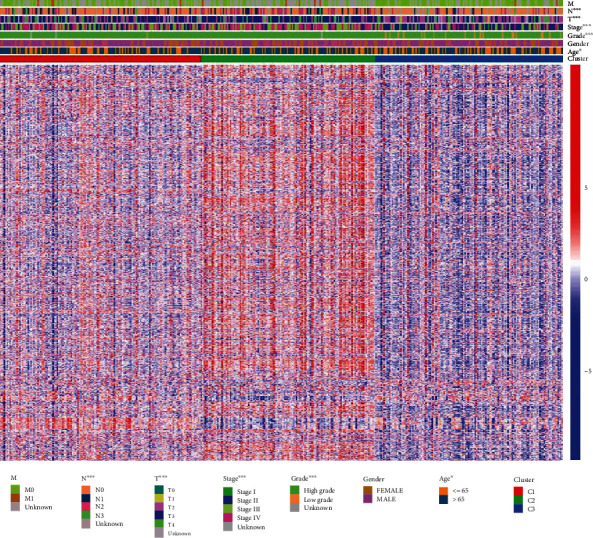
Heat map for gene sets with classified features.

**Figure 4:**
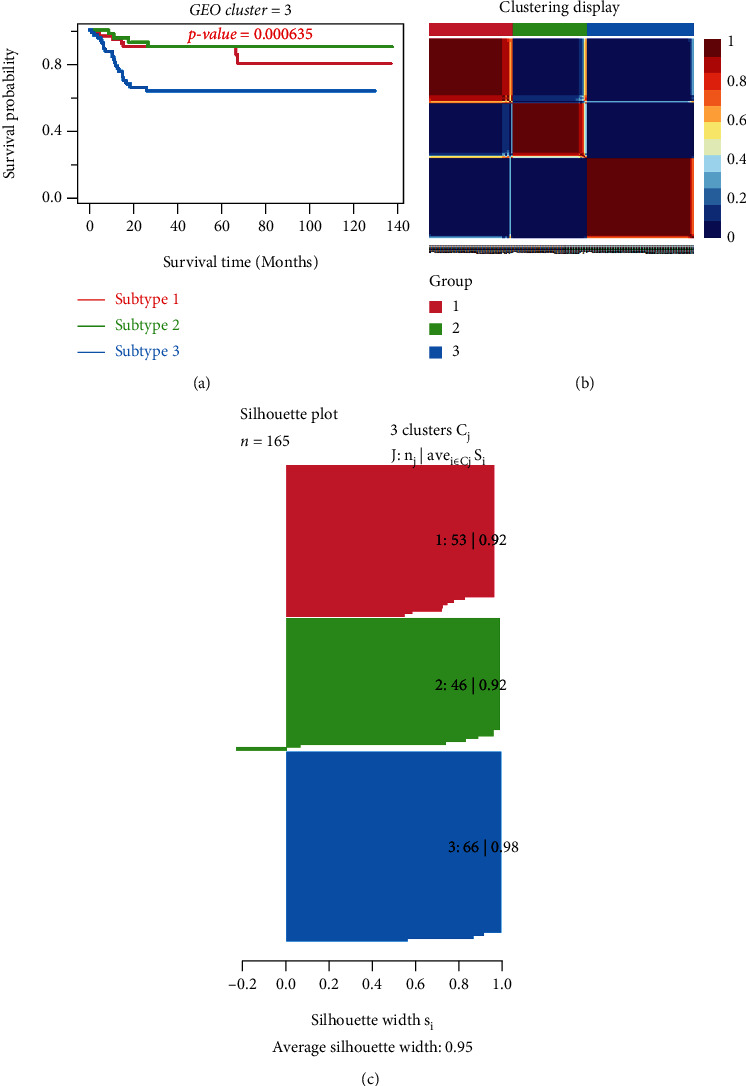
Classification of BLCA subtypes using “CancerSubtypes” in GEO. (a) Kaplan–Meier survival analysis of three subtypes. (b) Clustering heat map. (c) Average silhouette width represents the coherence of clusters.

The classification of BLCA was conducted through an unsupervised consensus clustering of the “CancerSubtypes” package in 414 tumor samples of TCGA. The samples with incomplete clinical information were discarded, leaving 407 samples. The *K* value determined the optimal number of clusters. In our study, the area under the cumulative distribution function (CDF) curve increased with no significance when *K* = 3; so, a three-cluster solution (*K* = 3) was chosen (Figure S1A). The results showed that 145 samples were cluster I matching subtype A, 126 were cluster II matching subtype B, and 136 were cluster III matching subtype C. Obviously, samples classified as subtype C had a better prognosis than samples classified as subtype A and subtype B (*P* = 0.00182). (Figure S1B; Figure 2). The clinical data show distinct characteristics of each BLCA subtype (Table 1), with significant age, grade, and stage differences. The heat map for gene sets with classified features shows how different gene sets express themselves across multiple subtypes (Figure 3). Similar classified results could be obtained when the GSE13507 dataset was analyzed similarly (Figure S2A-B; Figure 4). Based on the survival curve trend, cluster I matched subtype A, cluster II matched subtype C, and cluster III matched subtype B were significant (*P* < 0.001).

### 3.3. Identification of DEGSs

**Figure 5:**
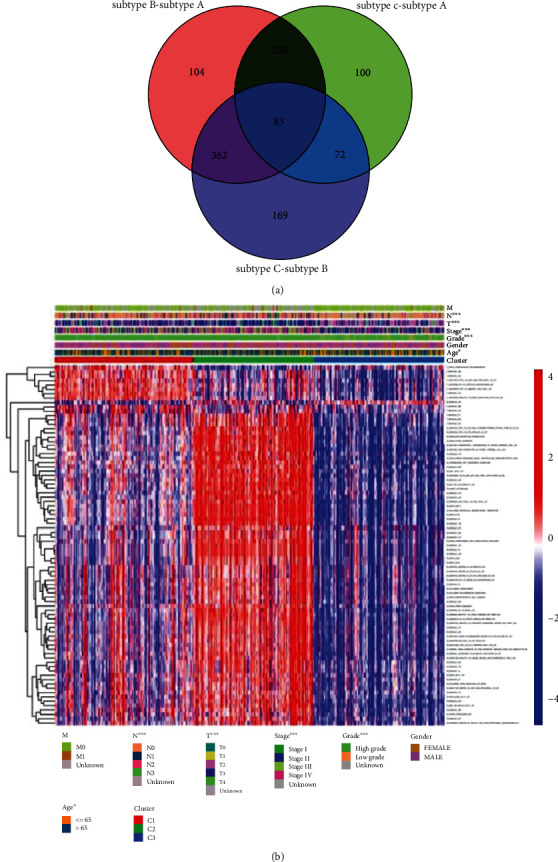
DEGSs among three subtypes. (a) Venn diagram of DEGSs. (b) Heat map for three subtypes with classified features.

We separately tested for the differential gene set expression among each subtype, including subtype C − subtype A, C − subtype B, and B − subtype A. Then, we compared the gene sets that were differentially expressed in these three groups. Finally, 83 DEGSs were obtained (Figure 5(a)), and their representation of these DEGSs in the three subtypes was markedly different, as shown in Figure 5(b).

### 3.4. Construction of Prognostic Signature

**Figure 6:**
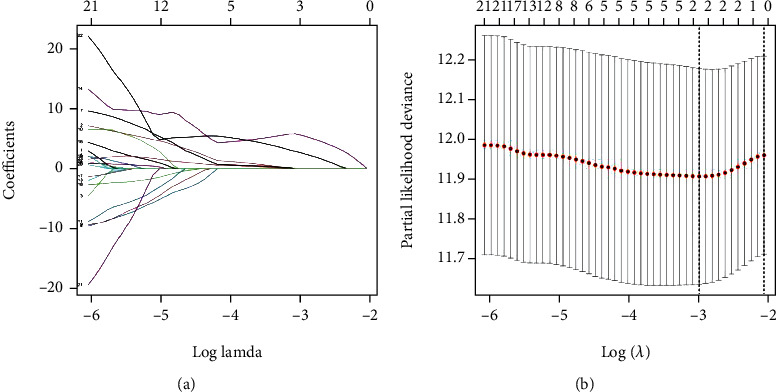
Establishment of BLCA prognostic signature. (a) Lasso coefficient profile of DEGSs with nonzero coefficients determined by the optimal lambda. (b) The crossvalidation of prognostic signature.

We applied the univariate cox regression model in 83 DEGSs to identify gene sets that influence patient's overall survival (OS). A total of 24 DEGSs were obtained (*P* < 0.05) (Table 2). After that, a Lasso regression model (Figure 6(a)) was established to reveal the log (Lambda) value of the 24 screened gene sets. And, after performing crossvalidation (Figure 6(b)), the gene sets with the slightest crossvalidation error were chosen. Finally, we discovered two DEGSs linked to prognosis: “GSE1460_ CD4_THYMOCYTE_ VS_THYMIC_STROMAL_CELL_DN” and “MODULE_253.”

### 3.5. Verification of Prognostic Signature Using TCGA and GEO Databases

The RS of BLCA samples in the TCGA was calculated, and the calculation formula used was RS = expression (GSE1460_CD4_THYMOCYTE_VS_THYMIC_STROMAL_CELL_DN)∗5.569 + expression (MODULE_253)∗2.604.

**Figure 7:**
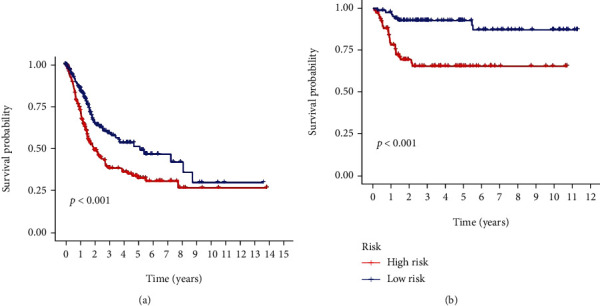
Kaplan–Meier survival analysis of prognostic signature. (a) The OS of the patients was worse in the high-RS group than in the low-RS group in the TCGA-BLCA cohort. (b) The results were validated using GSE13507.

**Figure 8:**
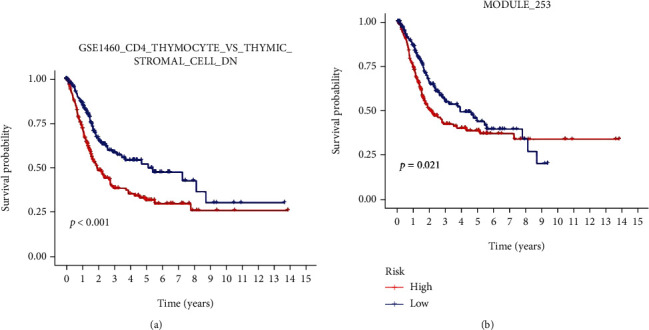
Kaplan–Meier survival analysis of prognostic-related DEGSs. (a) Survival analysis of “GSE1460_CD4_THYMOCYTE_VS_THYMIC_STROMAL_CELL_DN.” (b) Survival analysis of “MODULE_253”.

According to the median value of RS, the samples were divided into high- and low-RS groups. Kaplan–Meier survival analysis indicated that the OS of the patients was worse in the high-RS group than in the low-RS group in the TCGA-BLCA cohort (*P* < 0.001; Figure 7(a)). Similar results were confirmed using GSE13507 datasets in GEO databases (*P* < 0.001; Figure 7(b)). These results illustrate that the prognostic signature has effective predictive power in OS. Aside from that, we conducted survival analysis for the two DEGSs. The results suggested that having a high expression level of both DEGSs is associated with having a low OS (Figures 8(a) and 8(b)).

### 3.6. GO and KEGG of Prognostic Signature

**Figure 9:**
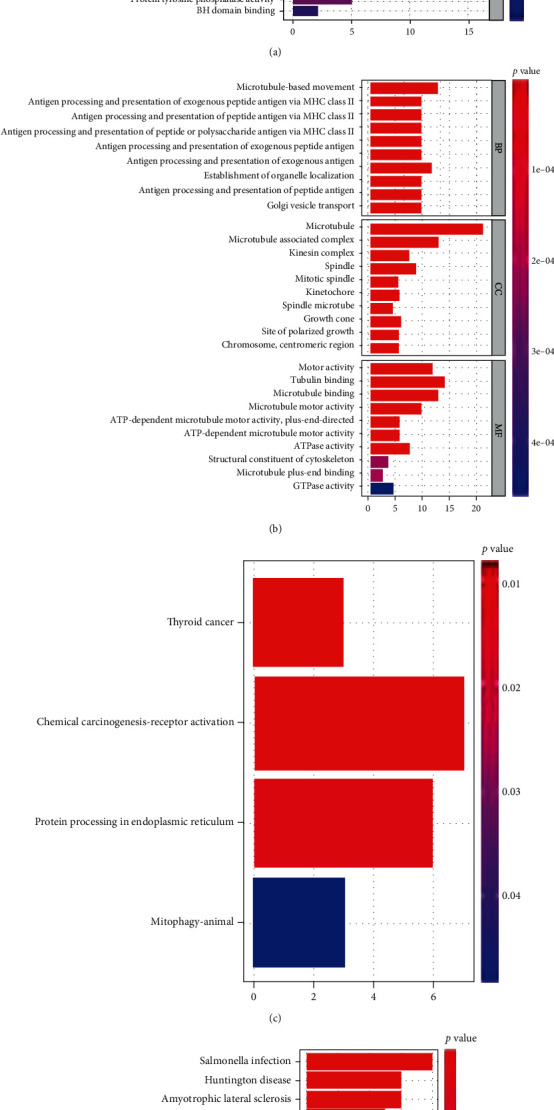
Gene functional enrichment analysis. (a) GO function enrichment of “GSE1460_CD4_THYMOCYTE_VS_THYMIC_STROMAL_CELL_DN.” BP: biological process; CC: cellular component; MF: molecular function. (b) GO function enrichment of “MODULE_253.” (c) KEGG analysis of “GSE1460_CD4_THYMOCYTE_VS_THYMIC_STROMAL_CELL_DN.” (d) KEGG analysis of “MODULE_253”.

Table 3 contains the gene list for two DEGSs. GO and KEGG analysis for “GSE1460_CD4_THYMOCYTE_VS_THYMIC_STROMAL_CELL_DN” (199 genes) and “MODULE_253” (21 genes) was performed, respectively. The GSEA database contains a detailed gene list. The former included 417 GO terms of biological process, 34 GO terms of cellular component, and 50 GO terms of molecular function (*P* < 0.05). The top 30 GO terms are shown in Figure 9(a). In addition, 234 GO terms of biological process, 64 GO terms of cellular component, and 31 GO terms of molecular function were discovered in the latter (*P* < 0.05). The top 30 significantly enriched GO terms are shown in Figure 9(b). Furthermore, genes from the former were enriched considerably into four KEGG pathways (*P* < 0.05; Figure 9(c)), while genes from the latter were significantly enriched into fifteen KEGG pathways (*P* < 0.05; Figure 9(d)).

### 3.7. PPI Network of Prognostic Signature

**Figure 10:**
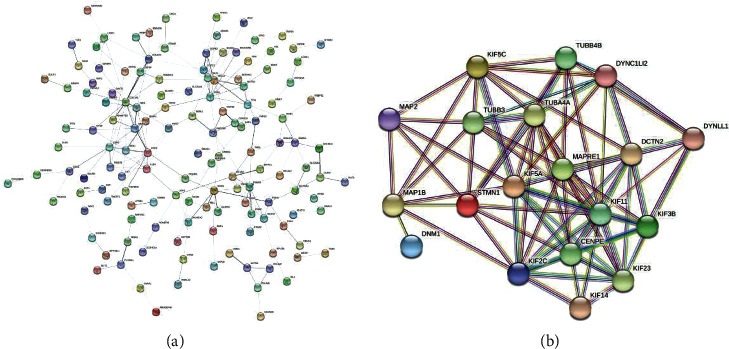
Protein-protein interaction network analysis. (a) Protein-protein interaction network analysis of “GSE1460_CD4_THYMOCYTE_VS_THYMIC_STROMAL_CELL_DN.” (b) Protein-protein interaction network analysis of “MODULE_253”.

**Figure 11:**
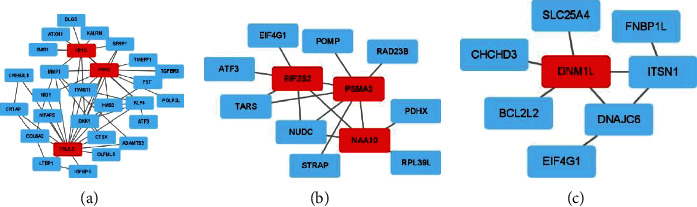
Subnet of “GSE1460_CD4_THYMOCYTE_VS_THYMIC_STROMAL_CELL_DN.” (a) Subnet 1. (b) Subnet 2. (c) Subnet 3.

**Figure 12:**
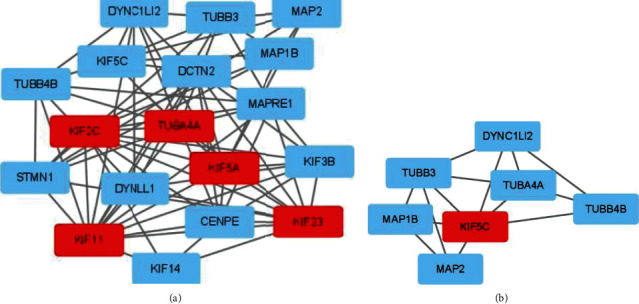
Subnet of “MODULE_253.” (a) Subnet 1. (b) Subnet 2.

**Figure 13:**
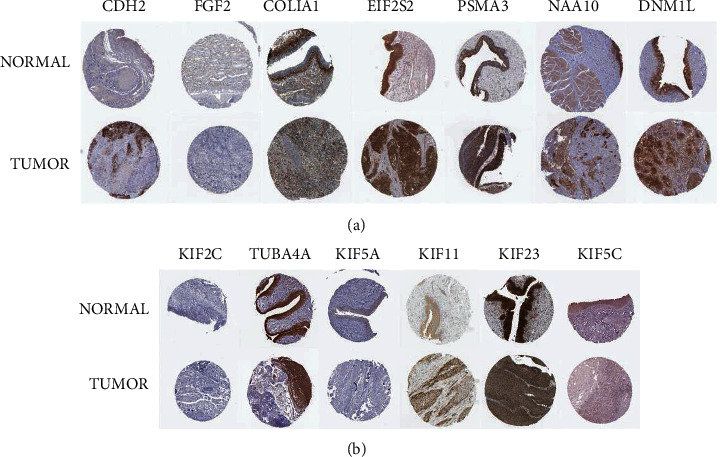
Verification of the hub protein expression using the HPA database. (a) Hub proteins of “GSE1460_CD4_THYMOCYTE_VS_THYMIC_STROMAL_CELL_DN.” (b) Hub proteins of “MODULE_253”.

The PPI network analysis was carried out separately for each DEGS via String software. We found a PPI network with 138 nodes and 184 edges from “GSE1460_CD4_THYMOCYTE_VS_THYMIC_STROMAL_CELL_DN” (Figure 10(a)) and a network with 19 nodes and 78 edges from “MODULE_253” (Figure 10(b)). In addition, for further analysis, we used the Cytotype software's MCODE app. The former's PPI network generated three functional subnet modules (subnet 1, subnet 2, and 3). The hub nodes, CDH2, FGF2, and COL1A1, had higher node degrees in subnet 1 (Figure 11(a)), EIF2S2, PSMA3, and NAA10 were hub nodes in subnet 2 (Figure 11(b)), and DNM1L was the hub node in subnet 3 (Figure 11(c)). Meanwhile, we obtained two functional subnet modules, namely, subnets 1 and 2. KIF2C, TUBA4A, KIF5A, KIF11, and KIF23 were filtered as hub nodes in subnet 1 (Figure 12(a)), while KIF5C was filtered as a node in subnet 2 (Figure 12(b)). To validate these findings, we referred to the HPA (https://www.proteinatlas.org/) database. We discovered that most of the proteins were observed in cancer-adjacent normal tissues and cancer tissues. Still, it was difficult to detect significant differences between normal and cancer tissue (Figures 13(a) and 13(b)).

### 3.8. Relation between Tumor-Infiltrating Immune Cells and RS

**Figure 14:**
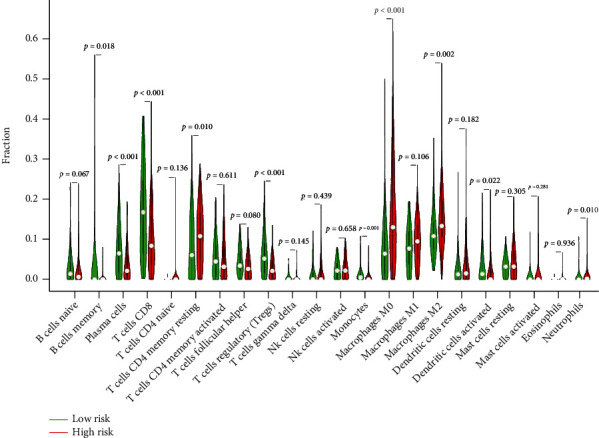
The composition of TIICs in BLCA samples.

The CIBERSORT algorithm was used to depict the composition of TIICs in all BLCA samples. As shown in the results, high-RS groups had higher fractions of CD8 T cells, M0 macrophages, M2 macrophages, activated dendritic cells, and neutrophils than low-RS groups. In contrast, low-RS groups had lower fractions of resting memory CD4 T cells, memory B cells, plasma cells, follicular helper T cells, regulatory T cells, and monocytes (Figure 14).

## 4. Discussion

Different BLCA subtypes have different invasive properties, which are also associated with different aggressiveness and prognoses [15]. We were able to identify DEGSs among multiple subtypes thanks to the help of GSVA analysis and the “Cancersubtypes” package. The “Cancersubtypes” package classified BLCA samples into three subtypes based on the gene set expression, and the prognosis differed significantly among the three subtypes. The significant differences in age, grade, and stage, on the other hand, may reflect the fact that the three subtypes of BLCA are at different stages of cancer progression. However, with the assistance of this advanced algorithm, we could still investigate the mechanism underlying BLCA progression. The combination of differential expression analysis and PPI network analysis can identify critical nodes influencing prognosis. Given the current state of treatment for BLCA, these critical nodes may be significant determinants of prognosis.

We discovered two DEGSs associated with prognosis in this study: “GSE1460_CD4_THYMOCYTE_VS_THYMIC_STROMAL_CELL_DN” and “MODULE_253.” Higher levels of both were linked to a lower OS rate. According to the GSVA database, the former is represented as differentially expressed genes between CD4 thymocytes and thymic stromal cells, implying that CD4+ T cells may play an essential role in BLCA progression, and the latter is defined as intracellular transport. We pursued this further to mine the biological significance. Following functional analysis, it was discovered that these DEGSs were primarily enriched in an extracellular matrix organization, MHC II antigen presentation, and the microtubule-associated pathway. This result was consistent with the GSVA database's description of DEGSs. The next step was to analyze the PPI network to find the corresponding proteins playing important roles. We analyzed both gene sets separately, resulting in several subnets and hub proteins. By comparing information about the protein expression in HPA, we discovered the following core proteins expressed in human BLCA tissue: CDH2, COL1A1, EIF2S2, PSMA3, NAA10, DNM1L, TUBA4A, KIF11, and KIF23.

CDH2, also known as N-cadherin, is a mesenchymal cell development regulator that was thought to be a discriminatory marker for interstitial cells in the human bladder and a critical biomarker of epithelial-mesenchymal transition (EMT) [16–18]. The EMT commonly thought to be a dysregulation of wound healing mechanisms is essential in cancer invasion and metastasis. The CDH2 expression is increasing, indicating a shift toward a mesenchymal phenotype. In summary, CDH2 may be involved in the progression of BLCA through the EMT. In subnet 1, COL1A1 (collagen type I alpha 1) is one of the hub nodes, along with CDH2. COL1A1 may also contribute to tumor progression by promoting EMT [19]. Many studies have shown that COL1A1 is a crucial factor in the invasion and metastasis of BLCA. The COL1A1 expression was higher in MIBC than in NMIBC, and the increased expression was associated with a poor prognosis in NMIBC.

Meanwhile, the low COL1A1 expression was thought to inhibit tumor proliferation and metastasis [19–21]. EIF2S2, PSMA3, and NAA10 were core proteins in “GSE1460_CD4_THYMOCYTE_VS_THYMIC_STROMAL_CELL_DN” subnet 2.

EIF2S2 is an RNA binding protein that regulates the gene expression. Its regulatory effects were reported to play a role in the occurrence and development of many cancers, but these did not involve BLCA. One of EIF2S2's carcinogenic pathways is through long noncoding RNA. EIF2S2 activated the Wnt signaling pathways to drive cancer development by regulating the interaction of LINC01600 with Myc protein. The other is glucose metabolism regulation. Typically, cancer cells rely on aerobic glycolysis (the Warburg effect) for energy, and knocking out EIF2S2 reduces the expression of glycolysis-related genes [22–24]. Although previous research has not addressed the carcinogenesis of BLCA, our findings suggest that EIF2S2 as a differential expression gene among three subtypes may play an essential role in BLCA development, which is supported by transcriptomics and proteomics analysis. PSMA3 participates in forming the 26S proteasome complex, which is involved in the degradation of several proteins [25]. There is currently no direct evidence that PSMA3 participates in tumor invasion and metastasis; however, some studies have suggested that the proteasome complex may play a role in cancer aggravation [26, 27]. PSMA3 was found to correlate with BLCA in our study, implying that we may have discovered an undiscovered biomarker that predicts BLCA progression, but further research is needed in follow-up studies. NAA10 catalyzes acetylation and is involved in cell proliferation [28]. More importantly, the NAA10 expression has been found in tumors from various organs, including the urinary bladder, cervix, liver, bone, lung, breast, colon, and prostate [29, 30]. However, the pathway by which NAA10 induces tumorigenesis varies depending on the target proteins in different cancer tissues. The NAA10–AR (androgen receptor) axis is essential for prostate cancer cell growth [31]. Considering the gender differences in BLCA epidemiology, we made the bold assumption that the NAA10–AR axis also promotes BLCA progression.

DNM1L, also known as dynamin-related protein 1, is a GTPase that functions in the cytosol of dynamins [32]. Mitochondria are the primary sites of cellular respiration, and their fragmentation is a typical phenotype in many cancers. DNM1L is an essential regulator of mitochondrial fission [33]. This could be why DNM1L has been linked to a poor prognosis.

The PPI network of “MODULE_253” is centered on TUBA4A, KIF11, and KIF23. They could be related to intracellular substance transport. TUBA4A (tubulin alpha 4a) is the gene that encodes -tubulin. TUBA4A mutations have been linked to neurodegenerative diseases such as amyotrophic lateral sclerosis (ALS) and frontotemporal dementia (FTD) [34, 35]. Little articles concern TUBA4A in cancer, and only lung cancer was mentioned [36–38]. TUBA4A could be a potential biomarker for BLCA prognosis prediction, but specific mechanisms must be investigated. The kinesin superfamily (KIFs) was a group of proteins that functioned as microtubule-based motors and served as the foundation for intracellular substance transport [39]. KIF11, also known as kinesin spindle protein, is important during mitogenesis and cell proliferation [40]. Furthermore, KIF11 is upregulated in various human cancers, including bladder cancer, renal clear cell carcinomas, prostate carcinomas, meningiomas, breast cancer, and gastric ccancer [41, 42]. Because high levels of the KIF11 expression indicate robust cell proliferation, anti-KIF11 therapy is emerging as a promising cancer therapy approach. KIF11 inhibitors were more effective at inhibiting the growth of gemcitabine-resistant bladder cancer cell lines [43]. Similarly, by regulating mitogenesis, KIF23 can influence cancer cell proliferation. Cell and animal experiments [44] confirmed the link between KIF23 and bladder cancer. According to the GSVA analysis and consensus clustering algorithm of “CancerSubtypes,” the high expression of gene set “GSE1460_CD4_THYMOCYTE_VS_THYMIC_STROMAL_CELL_DN” contributes to the poor prognosis of BLCA patients. As a result, we used the CIBERSORT algorithm to determine the composition of TIICs. We discovered that the fraction of resting memory CD4 T cells, memory B cells, plasma cells, follicular helper T cells, regulatory T cells, and monocytes was lower in the low-RS groups than in the high-RS groups. This finding suggests that these TIICs may play a role in antitumor immunity.

## 5. Conclusion

In this study, we identified two gene sets related to the prognosis of BLCA, and GO and KEGG analyses were performed based on the genes they contained. Further PPI analysis confirmed the network's core proteins as the most known therapeutic targets of BLCA or other cancers, while others were newly discovered biomarkers. In the HPA database, these proteins were partially validated. Given that BLCA is frequently multifocal, the tumor could have been embedded within the adjacent normal tissues. Further experimental validation will necessitate the collection of valuable donor transplant samples to rule out such effects.

## Figures and Tables

**Table 1:** The clinical information of BLCA.

Covariates	Cluster	Total	C1	C2	C3	*P* value
Age	<=65	160 (39.31%)	51 (35.17%)	44 (34.92%)	65 (47.79%)	0.0459
Age	>65	247 (60.69%)	94 (64.83%)	82 (65.08%)	71 (52.21%)	
Gender	Female	107 (26.29%)	39 (26.9%)	37 (29.37%)	31 (22.79%)	0.4723
Gender	Male	300 (73.71%)	106 (73.1%)	89 (70.63%)	105 (77.21%)	
Grade	High grade	383 (94.1%)	142 (97.93%)	125 (99.21%)	116 (85.29%)	0
Grade	Low grade	21 (5.16%)	1 (0.69%)	0 (0%)	20 (14.71%)	
Grade	Unknown	3 (0.74%)	2 (1.38%)	1 (0.79%)	0 (0%)	
Stage	Stage I	2 (0.49%)	0 (0%)	0 (0%)	2 (1.47%)	0
Stage	Stage II	130 (31.94%)	41 (28.28%)	17 (13.49%)	72 (52.94%)	
Stage	Stage III	140 (34.4%)	58 (40%)	48 (38.1%)	34 (25%)	
Stage	Stage IV	133 (32.68%)	45 (31.03%)	61 (48.41%)	27 (19.85%)	
Stage	Unknown	2 (0.49%)	1 (0.69%)	0 (0%)	1 (0.74%)	
T	T0	1 (0.25%)	0 (0%)	0 (0%)	1 (0.74%)	0
T	T1	3 (0.74%)	1 (0.69%)	0 (0%)	2 (1.47%)	
T	T2	119 (29.24%)	40 (27.59%)	20 (15.87%)	59 (43.38%)	
T	T3	193 (47.42%)	72 (49.66%)	81 (64.29%)	40 (29.41%)	
T	T4	58 (14.25%)	20 (13.79%)	23 (18.25%)	15 (11.03%)	
T	Unknown	33 (8.11%)	12 (8.28%)	2 (1.59%)	19 (13.97%)	
N	N0	237 (58.23%)	90 (62.07%)	59 (46.83%)	88 (64.71%)	8.00*E*-04
N	N1	46 (11.3%)	17 (11.72%)	20 (15.87%)	9 (6.62%)	
N	N2	75 (18.43%)	23 (15.86%)	38 (30.16%)	14 (10.29%)	
N	N3	7 (1.72%)	3 (2.07%)	2 (1.59%)	2 (1.47%)	
N	Unknown	42 (10.32%)	12 (8.28%)	7 (5.56%)	23 (16.91%)	
M	M0	196 (48.16%)	70 (48.28%)	37 (29.37%)	89 (65.44%)	0.0678
M	M1	11 (2.7%)	4 (2.76%)	5 (3.97%)	2 (1.47%)	
M	Unknown	200 (49.14%)	71 (48.97%)	84 (66.67%)	45 (33.09%)	

**Table 2:** Getting DEGSs were affecting patient's OS by the univariate cox regression model.

ID	HR	HR.95L	HR.95H	*P* value
MODULE_47	24.32049	3.300069	179.2345	0.001739
MODULE_234	36.33057	3.704609	356.2887	0.002041
HALLMARK_EPITHELIAL_MESENCHYMAL_TRANSITION	23.6657	3.364531	166.4617	0.001478
MODULE_122	19.64907	2.166845	178.1789	0.008112
KEGG_ECM_RECEPTOR_INTERACTION	85.10793	5.200854	1392.725	0.001832
GNF2_CDH11	17.45797	3.186617	95.64398	0.000982
MODULE_419	434.2375	12.08736	15599.95	0.000888
GSE6259_CD4_TCELL_VS_CD8_TCELL_UP	16777.27	78.41224	3589705	0.00038
ESC_V6.5_UP_EARLY.V1_DN	149.1306	3.299492	6740.413	0.010056
GSE1460_INTRATHYMIC_T_PROGENITOR_VS_THYMIC_STROMAL_CELL_DN	3467.784	45.39216	264925.2	0.000229
KEGG_ARRHYTHMOGENIC_RIGHT_VENTRICULAR_CARDIOMYOPATHY_ARVC	1561.694	15.57548	156585.1	0.001761
CAHOY_ASTROGLIAL	79.00986	2.572787	2426.38	0.012391
GSE4748_CTRL_VS_LPS_AND_CYANOBACTERIUM_LPSLIKE_STIM_DC_3H_UP	3983.537	19.1654	827980	0.002331
GSE1460_CD4_THYMOCYTE_VS_THYMIC_STROMAL_CELL_DN	31770.46	229.1448	4404910	3.80*E*-05
GSE4748_CTRL_VS_LPS_STIM_DC_3H_UP	4760.126	37.39764	605888.6	0.000616
CORDENONSI_YAP_CONSERVED_SIGNATURE	306.0277	10.18114	9198.675	0.000979
GSE3982_DC_VS_BCELL_UP	718.9345	3.175757	162753.9	0.017423
MODULE_385	28.34798	1.799017	446.6927	0.017436
PRC2_EZH2_UP.V1_UP	2167.861	16.44887	285710.8	0.00204
GSE36891_UNSTIM_VS_POLYIC_TLR3_STIM_PERITONEAL_MACROPHAGE_UP	75.70309	1.315493	4356.509	0.036387
MODULE_196	804.3902	11.05547	58527.01	0.002224
MODULE_253	1622.789	24.20709	108788.1	0.000571
MODULE_298	5.929103	1.155028	30.43587	0.032953
MODULE_153	5.274827	1.186505	23.45022	0.028916

**Table 3:** The list of genes of “GS460_CD4_THYM E1OCYTE_VS_THYMIC_STROMAL_CELL_DN” and “MODULE_253”.

GSE1460_CD4_THYMOCYTE_VS_THYMIC_STROMAL_CELL_DN	MODULE_253
GLIPR1	DNM1
NAV2	KIF5C
PON2	STMN1
AKR1B10	KIF11
CD59	MAPRE1
BAALC	ARSB
UAP1	KIF3B
LRIG1	MAP2
SLIT2	KIF14
BPNT2	KIF2C
NUDT15	TUBB3
SEPTIN2	TUBB4B
PTGS1	TUBA4A
COL1A1	SNTB2
NAA10	MAP1B
SS18	KIF23
ANXA3	CENPE
DUSP14	DYNLL1
COPZ2	KIF5A
SYNDIG1	DCTN2
BHLHE40	DYNC1LI2
ZC2HC1A	
PNPLA4	
NTAQ1	
KCNK1	
FARP1	
TPST1	
KIAA1549L	
MT1E	
DLG5	
COPB2	
STK3	
CTSK	
ATXN1	
RAD23B	
TWIST1	
SLC6A8	
LCMT1	
COPS7A	
SMUG1	
ELOVL1	
TMEM100	
TMEFF1	
EMC3	
CDC42BPA	
RRAS	
DENND5A	
DACT1	
CREG1	
CDH2	
BEX1	
SRPX2	
TFG	
DPY19L1	
BCL2L2	
SLC16A1	
MCFD2	
ACTN1	
CALR	
LARGE1	
GCLM	
NME7	
RBCK1	
TBK1	
HAS2	
POLR2L	
DHRS7	
TNFRSF12A	
EIF2S2	
ADAMTS3	
ADORA2B	
PCDH7	
TARS1	
UTP25	
RTL8C	
PLXNA1	
MMP1	
EMILIN1	
CRTAP	
DNAJC6	
DNM1L	
PDGFRL	
KLF4	
INA	
YARS2	
RECK	
MEIS3P1	
UST	
GULP1	
TLE1	
SLC39A7	
ASL	
ZBED8	
EOGT	
TMCO3	
DERL1	
ZCCHC24	
PDHX	
CPQ	
ARHGEF40	
TFPI	
PPP2R3A	
ATF3	
TFPI2	
SFRP1	
IPO7	
BNC2	
PF4V1	
MAP2K2	
SPA17	
SLC25A3	
TFE3	
GSTT2	
RABIF	
MYG1	
PHLDA1	
MEST	
RBP1	
TUBB2A	
BMERB1	
OLFML2A	
RPS6KA2	
NT5DC2	
COL6A2	
DOK5	
UCK2	
OLFML3	
RXRA	
KATNBL1	
BEX3	
IGFBP5	
TGFBR3	
RNF2	
KALRN	
ARMCX1	
DKK1	
TNFAIP1	
ANTKMT	
FGF2	
GAS1	
SRD5A1	
ADAM19	
POMP	
PHLDA3	
LIMS1	
TNFRSF10B	
SLC24A1	
SLC6A1	
CHCHD3	
BACE1	
SAP30	
TMA16	
SEC13	
SLC25A4	
CRYBG3	
SLC31A2	
MFAP5	
LDOC1	
ARHGAP29	
GYG2	
GLT8D2	
TRIM2	
NID1	
PDLIM5	
SAMD4A	
TENM4	
RRBP1	
PRRG1	
SIL1	
EIF4G1	
CREB3L1	
FNBP1L	
STAM2	
TSR3	
COPS6	
MLLT11	
STRAP	
C11orf24	
ITSN1	
EYA1	
SCG5	
PSMA3	
PTOV1	
FKBP9	
MTMR2	
PDLIM2	
LRRC15	
EXT1	
LTBP1	
FST	
SSH1	
C6orf120	
NUDC	
ME1	
HOMER3	
RPL39L	
ADGRL2	
COX17	
IQCK	

## Data Availability

The raw data can be obtained from public TCGA database (https://portal.gdc.cancer.gov/), GEO database (GSE13507) (https://www.ncbi.nlm.nih.gov/geo/), HPA database (https://www.proteinatlas.org/), and GSVA database (http://www.gsea-msigdb.org/gsea/index.jsp). Further inquiries can be directed to the corresponding author.

## Supplementary Materials

Supplementary 1 Figure S1: classification of TCGA samples by the consensus clustering algorithm. Several clusters *k*. (b) Cluster plot of BLCA.

Supplementary 2 Figure S2: classification of GEO samples by the consensus clustering algorithm. Many clusters *k*. (b) Cluster plot of BLCA.

## References

[1] Bray F., Ferlay J., Soerjomataram I., Siegel R. L., Torre L. A., Jemal A. (2018). Global cancer statistics 2018: GLOBOCAN estimates of incidence and mortality worldwide for 36 cancers in 185 countries. *CA: a Cancer Journal for Clinicians*.

[2] Cai Q., Chen Y., Xin S. (2020). Temporal trends of bladder cancer incidence and mortality from 1990 to 2016 and projections to 2030. *Translational Andrology and Urology*.

[3] Glaser A. P., Fantini D., Shilatifard A., Schaeffer E. M., Meeks J. J. (2017). The evolving genomic landscape of urothelial carcinoma. *Nature Reviews Urology*.

[4] Mariappan P., Johnston A., Padovani L. (2020). Enhanced quality and effectiveness of transurethral resection of bladder tumour in non-muscle-invasive bladder cancer: a multicentre real-world experience from Scotland's quality performance indicators programme. *European Urology*.

[5] Knowles M. A., Hurst C. D. (2015). Molecular biology of bladder cancer: new insights into pathogenesis and clinical diversity. *Nature Reviews Cancer*.

[6] Prout G. R., Barton B. A., Griffin P. P., Friedell G. H., National Bladder Cancer Group (1992). Treated history of noninvasive grade 1 transitional cell carcinoma. The National Bladder Cancer Group. *The Journal of Urology*.

[7] Willis D., Kamat A. M. (2015). Nonurothelial bladder cancer and rare variant histologies. *Hematology/Oncology Clinics of North America*.

[8] Sanli O., Dobruch J., Knowles M. A. (2017). Bladder cancer. *Nature Reviews Disease Primers*.

[9] Hanzelmann S., Castelo R., Guinney J. (2013). GSVA: gene set variation analysis for microarray and RNA-seq data. *BMC Bioinformatics*.

[10] Xu T., Le TD L. L., Su N. (2017). CancerSubtypes: an R/Bioconductor package for molecular cancer subtype identification, validation and visualization. *Bioinformatics*.

[11] Liu J., Tan Z., He J. (2020). Identification of three molecular subtypes based on immune infiltration in ovarian cancer and its prognostic value. *Bioscience Reports*.

[12] Song J., Deng Z., Su J., Yuan D., Liu J., Zhu J. (2019). Patterns of immune infiltration in HNC and their clinical implications: a gene expression-based study. *Frontiers in Oncology*.

[13] Wu M., Wang Y., Liu H., Song J., Ding J. (2020). Genomic analysis and clinical implications of immune cell infiltration in gastric cancer. *Bioscience Reports*.

[14] Szklarczyk D., Franceschini A., Wyder S. (2015). STRING v10: protein-protein interaction networks, integrated over the tree of life. *Nucleic Acids Research*.

[15] Pasquale V., Ducci G., Campioni G. (2020). Profiling and targeting of energy and redox metabolism in grade 2 bladder cancer cells with different invasiveness properties. *Cells*.

[16] Bhowmick N. A., Ghiassi M., Bakin A. (2001). Transforming growth factor-beta1 mediates epithelial to mesenchymal transdifferentiation through a RhoA-dependent mechanism. *Molecular Biology of the Cell*.

[17] Kuijpers K. A., Heesakkers J. P., Hafmans T. G., Schalken J. A. (2014). An update of the interstitial cell compartment in the normal human bladder. *BioMed Research International*.

[18] Lee J. M., Dedhar S., Kalluri R., Thompson E. W. (2006). The epithelial-mesenchymal transition: new insights in signaling, development, and disease. *The Journal of Cell Biology*.

[19] Zhu H., Chen H., Wang J., Zhou L., Liu S. (2019). Collagen stiffness promoted non-muscle-invasive bladder cancer progression to muscle-invasive bladder cancer. *Oncotargets and Therapy*.

[20] Brooks M., Mo Q., Krasnow R. (2016). Positive association of collagen type I with non-muscle invasive bladder cancer progression. *Oncotarget*.

[21] Ma H. P., Chang H. L., Bamodu O. A. (2019). Collagen 1A1 (COL1A1) is a reliable biomarker and putative therapeutic target for hepatocellular carcinogenesis and metastasis. *Cancers*.

[22] Yang J. W., Yuan L. L., Gao Y. (2021). 18F-FDG PET/CT metabolic parameters correlate with EIF2S2 expression status in colorectal cancer. *Journal of Cancer*.

[23] Yoshikawa N., Saito Y., Manabe H. (2019). Glucose depletion enhances the stem cell phenotype and gemcitabine resistance of cholangiocarcinoma organoids through AKT phosphorylation and reactive oxygen species. *Cancers*.

[24] Zhang J., Li S., Zhang L. (2020). RBP EIF2S2 promotes tumorigenesis and progression by regulating MYC-mediated inhibition via FHIT-related enhancers. *Molecular Therapy*.

[25] Kulichkova V. A., Fedorova O. A., Tsimokha A. S. (2010). 26S proteasome exhibits endoribonuclease activity controlled by extra-cellular stimuli. *Cell Cycle*.

[26] Alsamri H., El Hasasna H., Al Dhaheri Y., Eid A. H., Attoub S., Iratni R. (2019). Carnosol, a natural polyphenol, inhibits migration, metastasis, and tumor growth of breast cancer via a ROS-dependent proteasome degradation of STAT3. *Frontiers in Oncology*.

[27] Fan J., Du W., Zhang H. (2020). Transcriptional downregulation of miR-127-3p by CTCF promotes prostate cancer bone metastasis by targeting PSMB5. *FEBS Letters*.

[28] Wang Z., Wang Z., Guo J. (2012). Inactivation of androgen-induced regulator ARD1 inhibits androgen receptor acetylation and prostate tumorigenesis. *Proceedings of the National Academy of Sciences of the United States of America*.

[29] Kim S. M., Ha E., Kim J., Cho C., Shin S. J., Seo J. H. (2020). NAA10 as a new prognostic marker for cancer progression. *International Journal of Molecular Sciences*.

[30] Yu M., Gong J., Ma M. (2009). Immunohistochemical analysis of human arrest-defective-1 expressed in cancers in vivo. *Oncology Reports*.

[31] Kuhns K. J., Zhang G., Wang Z., Liu W. (2018). ARD1/NAA10 acetylation in prostate cancer. *Experimental & Molecular Medicine*.

[32] Jahani-Asl A., Slack R. S. (2007). The phosphorylation state of Drp1 determines cell fate. *EMBO Reports*.

[33] Ong S. B., Kalkhoran S. B., Hernandez-Resendiz S., Samangouei P., Ong S. G., Hausenloy D. J. (2017). Mitochondrial-shaping proteins in cardiac health and disease - the long and the short of it!. *Cardiovascular Drugs and Therapy*.

[34] Li J., He J., Tang L. (2015). TUBA4A may not be a significant genetic factor in Chinese ALS patients. *Amyotroph Lateral Scler Frontotemporal Degener*.

[35] Mol M. O., Wong T. H., Melhem S. (2021). NovelTUBA4Avariant associated with familial frontotemporal dementia. *Neurology Genetics*.

[36] Novikova S. E., Soloveva N. A., Farafonova T. E., Tikhonova O. V., Liao P. C., Zgoda V. G. (2021). Proteomic signature of extracellular vesicles for lung cancer recognition. *Molecules*.

[37] Pan D., Chen J., Feng C. (2019). Preferential localization of MUC1 glycoprotein in exosomes secreted by non-small cell lung carcinoma cells. *International Journal of Molecular Sciences*.

[38] Sun S., Guo W., Wang Z. (2020). Development and validation of an immune-related prognostic signature in lung adenocarcinoma. *Cancer Medicine*.

[39] Hirokawa N., Noda Y., Tanaka Y., Niwa S. (2009). Kinesin superfamily motor proteins and intracellular transport. *Nature Reviews Molecular Cell Biology*.

[40] Kapitein L. C., Peterman E. J., Kwok B. H., Kim J. H., Kapoor T. M., Schmidt C. F. (2005). The bipolar mitotic kinesin Eg5 moves on both microtubules that it crosslinks. *Nature*.

[41] Ding S., Xing N., Lu J. (2011). Overexpression of Eg5 predicts unfavorable prognosis in non-muscle invasive bladder urothelial carcinoma. *International Journal of Urology*.

[42] Mo X. C., Zhang Z. T., Song M. J. (2021). Screening and identification of hub genes in bladder cancer by bioinformatics analysis and KIF11 is a potential prognostic biomarker. *Oncology Letters*.

[43] Sun L., Lu J., Niu Z. (2015). A potent chemotherapeutic strategy with Eg5 inhibitor against gemcitabine resistant bladder cancer. *PLoS One*.

[44] Yao D. W., Song Q., He X. Z. (2021). Kinesin family member 23 (KIF23) contributes to the progression of bladder cancer cells in vitro and in vivo. *Neoplasma*.

