# Supplementary figures and images for: A Multiomics Profiling Based on Online Database Revealed Prognostic Biomarkers of BLCA
Source: Biomed Res Int. 2022 May 25;2022:2449449. doi: 10.1155/2022/2449449 (PMC9165618; doi:10.1155/2022/2449449)

**A**

Optimal number of clusters

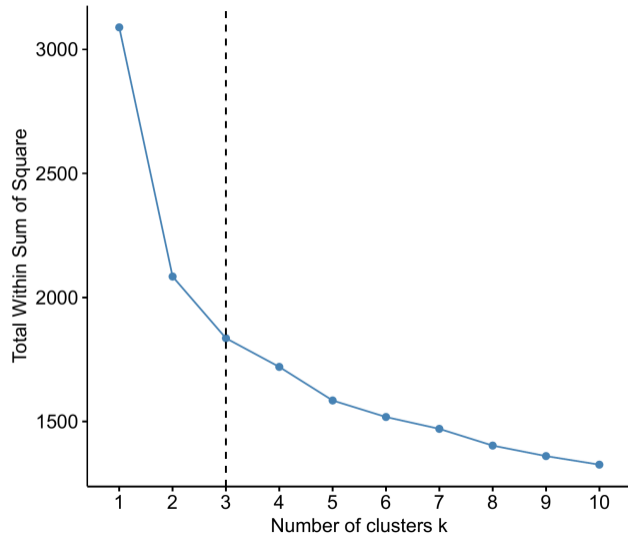**B**

Cluster plot

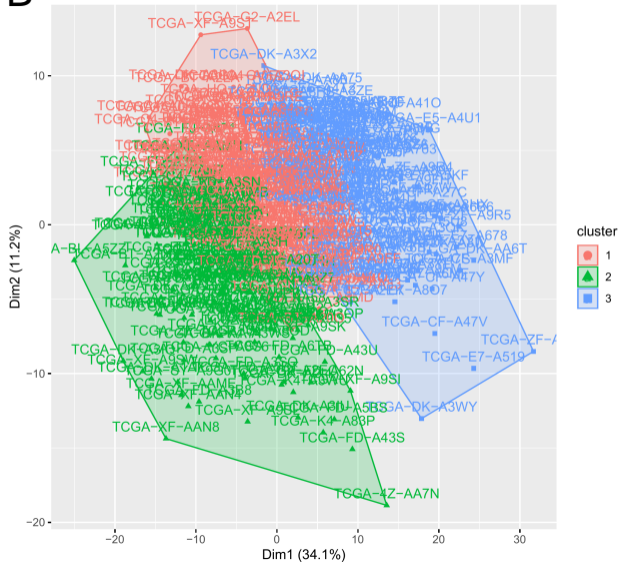

Supplement: Supplementary 1 — Figure S1: classification of TCGA samples by the consensus clustering algorithm. Several clusters k. (b) Cluster plot of BLCA. [file 2449449.f1.pdf]

**A**

Optimal number of clusters

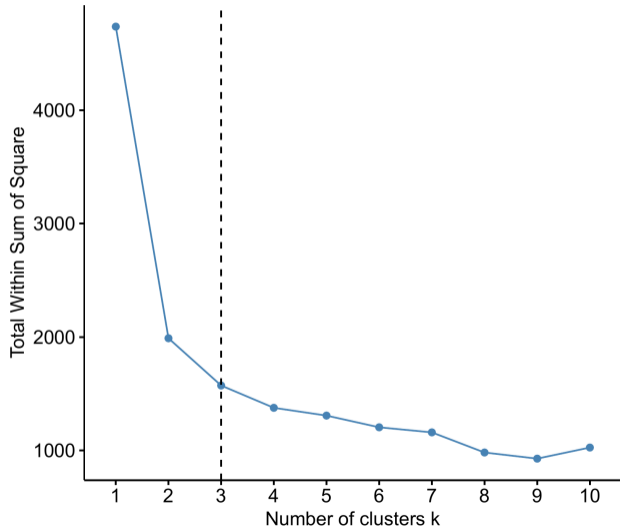**B**

Cluster plot

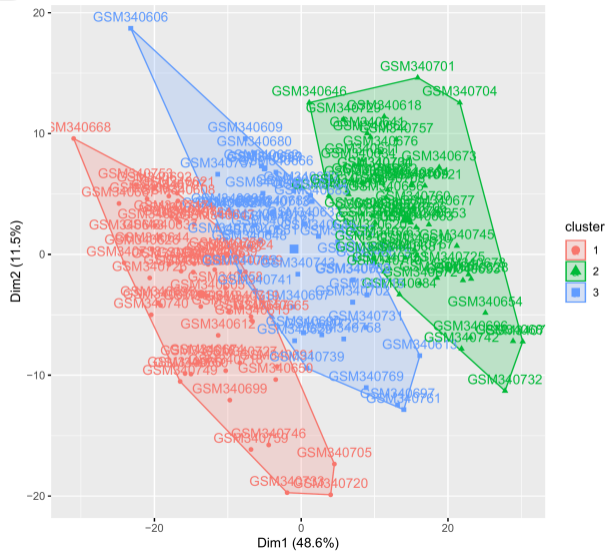

Supplement: Supplementary 2 — Figure S2: classification of GEO samples by the consensus clustering algorithm. Many clusters k. (b) Cluster plot of BLCA. [file 2449449.f2.pdf]
